# Depression severity and verbal comprehension in children and adolescents with a major depressive episode

**DOI:** 10.3389/fpsyt.2024.1395391

**Published:** 2024-09-24

**Authors:** Monia Trasolini, Giulia Serra, Maria Elena Iannoni, Elisa Andracchio, Massimo Apicella, Gino Maglio, Deny Menghini, Stefano Vicari

**Affiliations:** ^1^ Child & Adolescent Neuropsychiatry Unit, Bambino Gesù Children’s Hospital, IRCCS, Rome, Italy; ^2^ Department of Neuroscience, Università Cattolica del Sacro Cuore, Rome, Italy; ^3^ Department of Life Sciences and Public Health, Catholic University, Rome, Italy

**Keywords:** adolescent, depression, intelligent quotient, verbal comprehension, suicidal ideation, bipolar disorder, cognition

## Abstract

**Introduction:**

Severe depression is a prevalent psychiatric illness in children and adolescents associated with high levels of morbidity, disability, and a high risk of suicidal behavior. Cognitive factors associated with depression severity in juveniles have been poorly reported.

**Methods:**

We investigated the relationship between depression severity and intelligence quotient (IQ)with its subscales in 65 juveniles (aged 10–17 years) with a current major depressive episode evaluated at the Mood Disorder Program of Bambino Gesù Children’s Hospital in Rome. Pearson’s correlation analyses were followed by a Benjamini–Hochberg correction and linear multivariable regression model.

**Results:**

Depression severity measured with the total score of the Children’s Depression Rating Scale-Revised (CDRS-R) was positively associated with the Verbal Comprehension Index (VCI; Pearson’s r = 0.309 [0.042−0.534]; p = 0.024). The CDRS-R subscales positively associated with VCI by Pearson’s correlation were depressed feelings, suicidal ideation, excessive weeping, and reduced facial expressions. Suicidal ideation was the only factor independently and significantly associated with higher VCI in the multivariable linear regression model.

**Discussion:**

Suicidal ideation was significantly and independently associated with higher verbal comprehension, indicating that depressed juveniles with better verbal ability may be at a greater risk of showing suicidal ideation.

## 1 Introduction

Major depressive disorder (MDD) is a prevalent psychiatric disorder in children and adolescents, characterized by a stable and long-lasting alteration of emotions, energies, thoughts, and cognitive functions. This clinical condition has an impact on the quality of life of the affected participants, resulting in impairment of occupational and interpersonal functioning, with an increased risk of fatal outcomes. In 2008, the World Health Organization ranked major depression as the third cause of burden of disease worldwide and projected that it will rank first by 2030 (1); also, the Global Burden of Disease Study in 2010 identified depression as the second leading cause of years lived with disability (YLDs), accounting for approximately 10% of global YLDs and ranking among leading causes of school absence (2). The lifetime prevalence of MDD in a community sample of adolescents aged 12 to 18 years was estimated to be approximately 11%, more than a quarter of which (3%) is accounted for as severe depression (3). The prevalence of depression increases significantly from childhood to late adolescence, especially among women; it peaks at 13% for 15–17-year-old women, including 5% with severe depression. Severe depression is associated with a greater risk of psychiatric comorbidity, suicide, and functional impairment than the mild or moderate forms (3).

Psychopathological features of juvenile major depressive disorders include a) core symptoms of altered mood characterized by irritability, depressive feelings, mood lability, difficulty having fun, and lack of motivation; b) somatic symptoms like excessive fatigue, sleep and appetite disturbances, hypoactivity, listless speech and depressed facial affect, restlessness, and agitation; and c) cognitive/thought alterations including lack of concentration and attention with impairment of schoolwork, slow cognitive tempo, poor self-esteem or guilt rumination, catastrophic rumination, and suicidal ideation (4–7).

In child and adolescent psychiatry as in adult psychiatry, general intelligence and selective neuropsychological dysfunctions have been investigated in correlation with different psychiatric conditions, with an extensive amount of literature on mood disorders (8, 9) and schizophrenia (10).

Neuropsychological alterations in MDD are discretely characterized in adults: executive functions have been shown to be impaired during a depressive episode (11–13) along with attention and memory (8, 14–21), and a lower mean intelligence quotient (IQ) was reported in adult patients with MDD, particularly due to a lower working memory index (WMI) (22).

A meta-analysis showed significant moderate cognitive deficits in executive function, memory, and attention in adult patients with MDD relative to controls (23). Significant moderate deficits in executive function and attention and a non-significant trend toward small/moderate deficits in memory were found to persist in patients whose depressive symptoms had remitted, indicating that cognitive impairment in adults persists also after the remission of the acute depressive episode during the euthymic intervals (23). The neurobiological bases of this phenomenon are unclear, but some theories involving neuroinflammation, hypothalamic–pituitary axis activation, and its effects on neurotransmission in the prefrontal cortex (24) have been proposed.

Regarding adolescents, a longitudinal study with more than 20 years of follow-up showed that a lower IQ during late adolescence was associated with an increased risk of schizophrenia, severe depression, and other non-affective psychoses, but not bipolar disorder (25). However, studies on mood disorders are less numerous and tend to report less marked different profiles.

More recently (26), a meta-analysis of 17 studies compared 447 children and adolescents diagnosed with MDD and 1,347 healthy subjects on the presence and severity of cognitive deficits. They found that children and adolescents with MDD performed significantly lower than healthy controls in neuropsychological test procedures, and the most relevant deficits were found in inhibition capacity, phonemic verbal fluency, sustained attention, verbal memory, and planning.

Studies investigating the full-scale intelligence score and subscales in children and adolescents with MDD documented total lower performance and verbal IQ scores when compared with healthy controls (27–29). Moreover, a lower premorbid IQ has been described as a risk factor for later depression. However, this association is complexly mediated by sex and pubertal stage, and a paradoxical inversion of this association has been observed in adolescent subgroups when a higher IQ score was associated with a higher risk of depressive symptoms (30).

Studies investigating executive functions such as working memory, inhibition capacity, shifting, and planning ability found contradictory results. Indeed, some studies on working memory found that children and adolescents with depression showed lower performance than healthy subjects (31, 32), while others did not report such differences (33). Other studies found impairment in sustained attention (33, 34) but not in selective attention in children and adolescents with MDD (35).

Finally, among depressive and related disorders in children and adolescents, disruptive mood dysregulation disorder has been correlated with low normal IQ, with impairment of all considered indexes and adaptive abilities (36).

Relationships between cognitive functions and suicidal ideation and behaviors are even more complex. In adult studies, lower IQ has been described as a risk factor for suicide (37). This has been reported also on children and adolescents, but the evidence is conflicting (38). On adolescent inpatients, for instance, no correlation has been found between IQ and suicidal ideation or attempts. Of note, in another study, suicide attempts with high lethality correlated with more marked impairment of executive functioning in patients with MDD (39). A longitudinal study found more complex correlations between IQ and suicidal ideation or attempts in adolescents: although an increased risk of suicidal thoughts but not plans was related to lower IQ in men, higher IQ was associated with increased risk of non-suicidal self-harm in women (40). Conflicting results on cognitive deficits among juveniles diagnosed with MDD probably reflect the heterogeneity of different forms of juvenile depression themselves, ranging from mild full remitting transitory depressive episodes to severe forms of severe forms of Major Depressive Episodes (MDE) with high levels of morbidity, disability, and high risk of suicide (26).

To our knowledge, no studies have investigated the relationships between the severity of depressive episodes with suicidal ideation and cognitive functions. The aim of the present study is to investigate the relationship between depression severity and IQ with the Wechsler Intelligence Scale for Children Fourth Edition (WISC-IV) and its subscales in children and adolescents diagnosed with a MDE.

## 2 Methods

### 2.1 Participants

We analyzed neurocognitive factors associated with depression severity of 65 juveniles evaluated in the Mood Disorder Program at the Bambino Gesù Children’s Hospital IRCCS in Rome. First, participants were routinely screened in the general outpatient clinics and referred to the Day Hospital for assessment and treatment of early-onset mood disorders if the presenting illness was severe enough to require assessment and treatment in a specialized psychiatric program. In the present study, we included children and adolescents with a current primary or co-occurring clinically significant depressive symptomatology based on the Diagnostic and Statistical Manual of Mental Disorders, Fifth Edition (DSM-5) criteria without psychotic features and on significant scores on the well-validated Children’s Depression Rating Scale-Revised (CDRS-R T score ≥ 55, corresponding to a raw score of 30 or higher). We excluded patients with a diagnosis of autism spectrum disorder and patients with a diagnosis of substance-induced mood disorder and/or mood disorder due to another medical condition according to DSM-5.

Parents or legal representatives provided written informed consent for potential research analysis and anonymous reporting of findings in aggregate form, in accordance with Italian legal and ethical requirements for research uses of clinical data. The study has been conducted according to the guidelines established in the Declaration of Helsinki. Considering the retrospective nature of the analysis, the current study did not require the approval of the local ethics committee according to current legislation, but a notification was sent. Data were retrospectively analyzed in line with personal data protection policies.

### 2.2 Assessment

All study participants were assessed by an experienced child and adolescent psychiatrist and an experienced psychologist, using the Schedule for Affective Disorders and Schizophrenia for School-Age Children-Present and Lifetime Version (K-SADS-PL) (41). This semi-structured, clinician-administered diagnostic interview is performed with both subjects and their parents or adult legal representatives to assess current and past psychopathological features and psychiatric disorders in juveniles according to the criteria of the DSM-5 (4).

In addition, depressive and manic or mixed symptoms were rated by the same experienced clinicians using the CDRS-R (42) and the K-SADS Mania Rating Scale (KMRS) (43), respectively. Functional impairment was rated using the Children’s Global Assessment Scale (C-GAS) (44).

The CDRS-R (42) is a semi-structured interview used to rate depressive symptoms for ages 6–18 years on 17 items (rated 0 to 5 or 7) with raw total scores of 18–120 (given by the sum of the scores at single items and considered positive at scores >30, corresponding to a T-score >55). The CDRS-R included 17 subscales: school dysfunctions, difficulties in having fun, difficulties in interpersonal relationships, sleep disorders, appetite disorders, excessive fatigue, psychosomatic complaints, irritability, excessive guilt, low self-esteem, depressive feelings, morbid ideas, suicidal ideation, excessive crying, reduced facial expressions, slow speech, and motor hypoactivity. Total corrected (T-score) and single-item raw scores (0 to 7) were used for the present analyses.

KMRS (43) is a structured interview used to rate manic symptoms for ages 6–18 years on 14 items, with a total score of 1–68 [given by the sum of the scores (0–6) for single items minus 13 and considered positive at scores ≥12]. Symptoms rated in the KMRS are euphoria and expansiveness, irritability and anger, mood lability, reduced need for sleep, crowded thoughts, increased energy, increased activities, motor hyperactivity, grandiosity, rapid or pressured speech, distractibility, impaired judgment, hallucinations, and delusions. KMRS total scores of ≥12 are considered clinically significant, with subscale ratings of <2 considered normal, 2–3 borderline, and >3 as clinically significant. Raw scores (range 1–68) were used for the present statistical analyses.

IQ was measured using the WISC-IV (45) and the Wechsler Adult Intelligence Scale Fourth Edition (WAIS-IV) (46). The WISC-IV (6–16 years 11 months) and the WAIS-IV (16–90 years) are designed to assess cognitive ability. The core subtests are used to create four index scores that reflect different abilities, important in the expression of intelligent behavior in the classroom and beyond: Verbal Comprehension Index (VCI), Perceptual Reasoning Index (PRI), Working Memory Index (WMI), and Processing Speed Index (PSI). Standard scores (*mean* = 100; *standard deviation* = 15) for index scores and the full IQ were used in the statistical analyses.

### 2.3 Statistical analysis

We first analyzed the psychiatric symptoms (CDRS-R total score, KMRS score, and C-GAS score) associated with cognitive abilities (full IQ, VCI, PRI, WMI, and PSI) by performing Pearson’s correlation analyses.

As VCI was found to be significantly associated with total CDRS-R T score, we further analyzed the correlation between VCI and single CDRS-R items’ scores by performing Pearson’s correlation analyses.

To reduce the possibility of type I error, we applied a Benjamini–Hochberg correction (47) to multiple testing with a false discovery rate set at 0.05. Finally, we applied a linear multivariable regression model to test for factors independently and significantly associated with VCI.

Averages are reported as means with standard deviation (± SD) or 95% confidence interval (CI). Analyses were made with commercial statistical software (Stata.13^®^; StataCorp, College Station, TX, USA) using data spreadsheets based on Excel (Microsoft Corp., Redmond, WA, USA) and Statview.5^®^ (SAS Institute; Cary, NC, USA) software.

## 3 Results

### 3.1 Sample characteristics

We included 65 subjects with an average age of 15.0 [CI 14.5–15.5] years (age range 10–17 years); 58.5% were men diagnosed with current primary or co-occurring MDE.

Categorical diagnoses following the DSM-5 diagnostic criteria (4) were as follows: MDD (53.8%), bipolar disorder (21.5%), or depressive disorder not otherwise specified (NOS) (24.6%), co-occurring with other psychiatric conditions including anxiety and attention deficit hyperactivity disorder (ADHD).

All subjects were diagnosed with current primary or co-occurring clinically significant depressive symptomatology as rated with a CDRS-R total T-score of 69.5 [CI 67.4–71.7]. Manic symptoms rating was 8.42 [CI 6.56–10.8], and global functioning was significantly compromised for all subjects as rated with an averaged C-GAS total score of 46.3 [CI 44.4–48.1]. Twenty-seven patients (38%) reported at least one suicide attempt lifetime.

Cognitive abilities as rated with WISC-IV and WAIS-IV were as follows: full IQ 98.2 [CI 92.4–103.9], VCI 103 [CI 96.7–109], PRI 104 [CI 98.6–109], WMI 92.5 [CI 88.5–96.6], and PSI 91.1 [CI 86.3–96.0] (**Table 1**).

**Table 1:** Characteristics of the sample.

Factor	Percent or average [95% CI]
Age	15.0 [14.5–15.5]
*Sex* Male Female	58.5 [46.9–70.9] 41.5 [29.2-53.1]
*Diagnosis* MDD Bipolar Disorder Depressive Disorder–Not Otherwise Specified	53.8 [41.3–66.7] 21.5 [12.3–31.8] 24.6 [14.5–35.4]
Total IQ	98.2 [96.7–103]
VCI	103 [96.7–109]
PRI	104 [98.6–109]
WMI	92.5 [88.5–96.6]
PSI	91.1 [86.3–96.0]
CDRS-R total T-score	69.5 [67.4–71.7]
KMRS total score	8.42 [6.56–10.8]
C-GAS score	46.3 [44.4–48.1]

MDD, Major Depressive Disorder; IQ, intelligence quotient; VCI, Verbal Comprehension Index; PRI, Perceptual Reasoning Index; WMI, Working Memory Index; PSI, Processing Speed Index; CDRS-R, Children's Depression Rating Scale-Revised; KMRS, Kiddie Schedule for Affective Disorders and Schizophrenia Mania Rating Scale; C-GAS, Children’s Global Assessment Scale.

### 3.2 Factors associated with cognitive abilities

Depression severity score (CDRS-R T-score) was associated with VCI (Pearson’s r = 0.309 0.309 [0.042−0.534]; *p* = 0.024), but not with full IQ (Pearson’s r = −0.213; *p* = 0.126) and with the other index scores including PRI (Pearson’s r = 0.037; *p* = 0.792), WMI (Pearson’s r = 0.256; *p* = 0.064), and PSI (Pearson’s r = 0.057; *p* = 0.684).

Depression severity score (CDRS-R T-score) also appears to be associated with the absolute difference between VCI and IQ (Pearson’s r = 0.237; *p* = 0.047). This correlation loses significance when correction for multiple comparisons is applied.

Manic symptoms’ scores rated with KMRS and global functioning scores rated with C-GAS did not correlate with any cognitive ability score. Each cognitive ability score did not significantly differ between sex, age, and diagnosis.

### 3.3 Depressive symptoms associated with verbal comprehension score

CDRS-R single item scores associated with VCI were depressed feelings (Pearson’s r = 0.332; *p* = 0.007), suicidal ideation (Pearson’s r = 0.365; *p* = 0.003), excessive weeping (Pearson’s r = 0.248; *p* = 0.048), and Depressed facial affect (Pearson’s r = 0.266; *p* = 0.033). After applying a Benjamini–Hochberg correction, only depressed feelings and suicidal ideation were still significant (**Table 2**).

**Table 2:** Depressive symptoms (CDRS-R items) associated with VCI.

Depressive symptoms (CDRS-R items scores)	Pearson’s r correlation [95% CI]	*p*-Value
Impaired school work	−0.127 [−0.362 to 0.122]	0.317
Difficulty having fun	0.174 [−0.075 to 0.402]	0.170
Social withdrawal	−0.044 [−0.287 to 0.204]	0.729
Sleep disturbance	0.145 [−0.104 to 0.378]	0.252
Appetite disturbance	−0.067 [−0.307 to 0.182]	0.601
Excessive fatigue	0.034 [−0.213 to 0.278]	0.789
Physical complaints	−0.074 −0.314 to 0.175]	0.561
Irritability	0.168 [−0.082 to 0.397]	0.186
Excessive guilt	0.121 [−0.128 to 0.356]	0.340
Low self-esteem	0.109 [−0.140 to 0.346]	0.390
Depressed feelings^a^	0.332 [0.093 to 0.534]	0.007
Morbid ideation	0.029 [−0.218 to 0.273]	0.817
Suicidal ideation^a^	0.365 [0.131 to 0.561]	0.003
Excessive weeping	0.248 [0.003 to 0.466]	0.048
Depressed facial affect	0.266 [0.022 to 0.481]	0.033
Listless speech	0.000 [−0.246 to 0.246]	0.999
Hypoactivity	0.100 [0.150 to 0.337]	0.433

CDRS-R, Children’s Depression Rating Scale-Revised; VCI, Verbal Comprehension Index.

a Significance after the Benjamini–Hochberg (BH) correction (p < BH).

We applied a linear multivariable regression model to test for CDRS-R single-item scores independently and significantly associated with VCI. Only suicidal ideation resulted in being significantly and independently associated with VCI (β = 0.360, t = 3.16 [CI 1.45–6.44], *p* = 0.002) after controlling for CDRS-R total score.

## 4 Discussion

This study analyzed the association between the severity of depressive episodes with IQ and its subscales (VCI, PRI, WMI, and PSI) in 65 juvenile patients diagnosed with current MDEs.

This sample was considered to have clinically significant depressive symptoms rated with a CDRS T–score of ≥55, with an average score of 69.5 [67.4–71.7]. Study subjects also had high rates of global functional impairment (C-GAS score 46.3 [44.4–48.1]) (**Table 1**). Slightly less than a quarter of participants met the criteria for a bipolar disorder diagnosis, consistent with the wide range of literature reporting that pediatric-onset MDEs have a high risk of progressing to bipolar disorder (48, 49). It is also worth considering that a significant percentage of patients in the MDD and depressive disorder–NOS groups may be diagnosed with bipolar disorder at follow-up when more episodes in the clinical history may allow reaching DSM-5 criteria for bipolar disorder diagnosis.

Individuals in our sample had normal mean IQ scores (98.2 [96.7–103]), and they showed a trend toward better performance in VCI (103 [96.7–109]) and in PRI (104 [98.6–109]) than WMI (92.5 [88.5–96.6]) and PSI (91.1 [86.3–96.0]) (**Table 1**). In agreement with previous studies, our results show that children and adolescents with depression have mainly lower scores on measures of working memory (50) and processing speed than they do on measures of verbal intelligence (36, 51, 52). It has been suggested that children and adolescents with depression perform lower in working memory and processing speed tests because they are less able to focus their attention on the test due to depressive symptoms like rumination (53).

Investigating demographic and psychopathological factors associated with the cognitive abilities of our patients, we found that depression severity score was associated with higher VCI of WISC-IV (Pearson’s r = 0.309 [0.042−0.534]; *p* = 0.024, **Table 2**). The VCI is composed of subtests (Vocabulary, Similarities, and Comprehension) measuring verbal abilities utilizing reasoning, comprehension, and conceptualization (45). Patients with a better ability to listen to a request, retrieve previously learned information, formulate verbal concepts, and express the answer verbally seem to show more severe depressive symptoms.

A first interpretation of this result could be that children and adolescents with stronger verbal abilities may be more able to transform their feelings and thoughts into spoken words than children and adolescents with weaker verbal abilities. However, previous studies that investigated the relationship between depressive rumination and intelligence support a more profound and direct correlation between verbal intelligence and depressive feelings.

Rumination is a trans-diagnostic emotion regulation strategy that has been associated with various forms of psychopathology such as depression, anxiety, substance abuse, binge eating, and self-injurious behavior (54). Depressive rumination has been conceptualized as repetitive thinking about the symptoms, causes, circumstances, meanings, implications, and consequences of depressed mood and distress, as outlined in the Response Styles Theory (55).

Individuals with a ruminative response to depression show higher dysfunctional beliefs and faulty attributions of negative events to themselves and the perception that these events are global and stable (56).

Studies have demonstrated that verbal intelligence is a predictor of rumination severity, so a ruminating mind is a more verbally intelligent mind (57).

Furthermore, it is well-established that rumination increases depressive symptoms (58, 59), so individuals with a ruminative response to depression are prone to having more severe and prolonged depressive episodes (56, 60). Also, rumination can be considered a predictor of the onset and reoccurrence of MDEs (59, 61). Moreover, from a neurobiological point of view, rumination, depression, and high intelligence may all correlate with brain network over-connectivity (62).

As a further step to test depressive symptoms associated with verbal comprehension scores, we constructed a multivariable linear regression model. Among all depressive symptoms measured using the CDRS-R, only suicidal ideation resulted to be significantly and independently associated with VCI, indicating that children and adolescents with a better verbal skills may be at greater risk of showing suicidal ideation independently from the severity of the depressive episode.

Previous studies have shown that children who had higher VCI scores were judged by parents to be "more suicidal" than children who scored lower on these measures of cognitive functioning (50). Also, preadolescents who were more suicidal had greater verbal, performance, and total IQ scores than their non-suicidal counterparts (63). Once again, this relationship between stronger verbal abilities and suicidal ideation may be related to the ability of children and adolescents with higher VCI to transform suicidal thoughts into spoken words than peers with weaker verbal abilities. Moreover, recent literature reports that rumination may be a potential cognitive factor related to suicide and was shown to be a good predictor of the presence and duration of suicidal ideation (64–66).

As mentioned above, individuals with a passive style, focusing on their depressive symptoms, experiencing an incessant barrage of negative thoughts, and responding to negative effects with rumination, may be at a greater risk of worsening and persistence of affective symptoms (55) and increased hopelessness (67). The severity of depressive symptoms and psychological indicators such as hopelessness are considered predictors of suicidal ideation (68, 69). Furthermore, this type of thinking may interfere with the ability to adaptively regulate affects and mood, with deficits in mood regulation also strongly linked to suicidal ideation (70, 71).

Most of the studies correlating rumination and severity of depressive symptoms and suicidal ideation were conducted on adult samples; few studies on adolescents suggest that rumination in response to negative affect may increase the risk of experiencing suicidal thoughts also in this age group (64).

Conversely, individuals admitted to the inpatient unit of our same institution and diagnosed with Dysruptive Mood Dysregulation Disorder have been reported to have a lower IQ, including lower VCI and PRI, compared to patients with MDD (36).

Whereas high general intelligence (g) is considered a protective factor for MDD (72) and is predictive of successful coping and resilience (73), it has also been reported that the association between intelligence and better mental health outcomes is not linear, particularly considering patients with highest levels of IQ (74). Some authors proposed different theories to explain how high intelligence may paradoxically represent a risk factor for developing affective disorders (75). Indeed, the conceptualization of over-excitability (75) suggests that intellectually gifted individuals may overreact to stimuli, and this may be linked to anxiety and depression. Such over-excitability seems to correlate with chronic hypothalamic–pituitary–adrenal activation (74), a well−studied neurobiological substrate of depression (76) and suicidal behaviors (77). Patients in our cohort had an IQ distribution comparable to the general population (in our sample: 5th pc = 74, 10th pc = 77.2, 25th pc = 85.5, 50th pc = 98, 75th pc = 113.5, 90th pc = 125.4, and 95th pc = 131.7), with only six patients with an IQ above 125 and no patients with IQ < 70. While the over-excitability theory is interesting in explaining the depression and intelligence correlation in “gifted” children, probably this accounts only for a minor part of the correlation observed in our sample.

Sub-syndromic and under-recognized bipolar disorder may be another important factor linking depression severity with high verbal intelligence in our sample. Indeed, many studies reported that high verbal intelligence is correlated with bipolar disorder diagnosis (78–81). A high proportion of participants in our cohort are diagnosed with bipolar disorder, and another 10%–20% of the sample may progress to bipolar disorder during late adolescence or early adulthood (48, 49). Also, bipolar disorder is associated with a greater suicidal risk than MDD during both adult and pediatric age (82). We speculate that a correlation between pediatric bipolar disorder, high verbal intelligence, and suicidal ideation may be the key to interpreting the present findings and needs to be explored in further studies.

Lastly, it is worth mentioning that depressed individuals in our sample have a mainly discrepant cognitive profile with VCI and PRI of approximately 10 points higher than “fluid” intelligence indexes (WMI and PSI). This discrepancy in the IQ indexes on depressed subjects is coherent with previous observations on adults (14, 22, 83) and adolescents diagnosed with depressive disorders admitted to our inpatient ward for acute psychopathology (36) and is probably a consequence rather than a risk factor for depression, as we learn from recent appropriately designed longitudinal studies (84). This point underlies that depressed individuals in our sample suffer from a serious depressive episode burdened by relevant cognitive cluster symptoms. Our results contribute to implementing knowledge on cognitive functions of children and adolescents diagnosed with severe depressive symptoms and suggest that assessment of maladaptive response styles to negative affect may be important to evaluate suicidal ideation.

The main strength of our work is that we included carefully selected patients for homogeneity of current active psychopathology and with an IQ distribution comparable to the one of the general population (with the exclusion of patients with intellectual disabilities). The present results should be interpreted in light of some limitations including the cross-sectional design that does not allow to make inferences on causality. Further studies should explore whether rumination mediates causally the correlation between VCI and depression and whether discrepant cognitive profiles observed are a risk factor for or a consequence of major depression. Also, we did not include specific measures for executive functions. Some children may have high intelligence but marked executive function deficits, with neuropsychological profiles that can decrease their potential (85, 86). This exploratory study should be interpreted as a first step to designing appropriate longitudinal studies to understand relationships between cognitive abilities, depression severity, and suicidal ideation in pediatric populations.

## Data Availability

The datasets presented in this article are not readily available because of the nature of the research, due to ethical reasons. Further inquiries can be directed to the corresponding author.
